# Sample size determination for bibliographic retrieval studies

**DOI:** 10.1186/1472-6947-8-43

**Published:** 2008-09-29

**Authors:** Xiaomei Yao, Nancy L Wilczynski, Stephen D Walter, R Brian Haynes

**Affiliations:** 1 Department of Clinical Epidemiology and Biostatistics, McMaster University, Hamilton, Ontario, Canada

## Abstract

**Background:**

Research for developing search strategies to retrieve high-quality clinical journal articles from MEDLINE is expensive and time-consuming. The objective of this study was to determine the minimal number of high-quality articles in a journal subset that would need to be hand-searched to update or create new MEDLINE search strategies for treatment, diagnosis, and prognosis studies.

**Methods:**

The desired width of the 95% confidence intervals (W) for the lowest sensitivity among existing search strategies was used to calculate the number of high-quality articles needed to reliably update search strategies. New search strategies were derived in journal subsets formed by 2 approaches: random sampling of journals and top journals (having the most high-quality articles). The new strategies were tested in both the original large journal database and in a low-yielding journal (having few high-quality articles) subset.

**Results:**

For treatment studies, if W was 10% or less for the lowest sensitivity among our existing search strategies, a subset of 15 randomly selected journals or 2 top journals were adequate for updating search strategies, based on each approach having at least 99 high-quality articles. The new strategies derived in 15 randomly selected journals or 2 top journals performed well in the original large journal database. Nevertheless, the new search strategies developed using the random sampling approach performed better than those developed using the top journal approach in a low-yielding journal subset. For studies of diagnosis and prognosis, no journal subset had enough high-quality articles to achieve the expected W (10%).

**Conclusion:**

The approach of randomly sampling a small subset of journals that includes sufficient high-quality articles is an efficient way to update or create search strategies for high-quality articles on therapy in MEDLINE. The concentrations of diagnosis and prognosis articles are too low for this approach.

## Background

For clinicians and clinical researchers, it is important to be able to quickly retrieve articles that are clinically sound and directly relevant without missing key studies or retrieving excessive numbers of preliminary, irrelevant, outdated, or misleading reports. Unfortunately, reliable and precise retrieval of clinical articles from MEDLINE is not easy because of the size of the database (> 5000 journals published in 37 languages and > 10,000 citations added each week [1,2]) and the limitations of indexing. Although indexers are trained by National Library of Medicine, the inter-indexer consistency for duplicate indexing of the same article is quite low, ranging from 0.3 to 0.6 [3,4].

One possible solution to this problem is to develop methodological search filters or strategies to retrieve original studies and review articles that use the strongest methods to assess clinically important problems [5,6]. For instance, randomized trials provide the strongest test of therapeutic interventions. The Hedges Team in the Health Information Research Unit at McMaster University has been developing search strategies for some time, for retrieving high-quality articles (passing our methodological criteria) on treatment [7], diagnosis [8], prognosis [9], etiology [10], clinical prediction guides [11], systematic reviews [12], and qualitative studies [13] from MEDLINE based on a database with over 49,000 articles from 161 clinical journals published in 2000. These strategies, which all focus on clinical applications, have been adopted for use in the Clinical Queries interface of PubMed  and also in Ovid.

The Clinical Queries search strategies were developed using index terms and text words available in the year 2000. Periodic updating of search strategies is necessary because index terms used in MEDLINE are updated annually as new concepts emerge, some old concepts fall out-of-date, and new journals are added [14]. New search strategies are also needed for purposes not covered by the existing search strategies. However, the development and testing of the Clinical Queries search strategies in a 161-journal database was highly labor-intensive and expensive. Six research assistants on the Hedges team devoted 1 day per week over a 14 month period for calibration and 1 day per week over a 12 month period for data collection.

In this paper, we set out to determine the least number of high-quality articles in a journal subset that would need to be hand-searched to update the search strategies for retrieving studies of treatment, diagnosis, and prognosis (the 3 most important clinical categories) from MEDLINE.

## Methods

### Data organization

Hand searching of the literature provided a "gold standard" classification of article categories. Six research assistants assessed 49,028 articles from 161 journals published in 2000 that were indexed in MEDLINE; all the articles were classified as original studies, review articles, general papers, or case reports; and the original and review articles were then categorized as "pass" or "fail" studies based on methodological criteria for treatment, diagnosis, prognosis, and other clinical topic areas [15]. Article citations downloaded from MEDLINE were matched with the hand-searched data. Index terms and textwords related to research design features indicating methodologic rigor were treated as "diagnostic tests" for retrieving "pass articles" – high-quality studies.

The sensitivity (the proportion of the relevant and sound articles that had been found in hand-searched journals that were detected by a given search strategy), specificity (the proportion of irrelevant and poor-quality studies that were excluded by the search strategy), precision (the proportion of retrieved articles that were relevant and sound), and accuracy (the proportion of all articles that were correctly classified) for each single term and combinations of terms were determined by using an automated iterative process. All combinations of search terms used the Boolean "OR", meaning that articles that included any one of the search terms in the strategy would be retrieved. Search strategies were developed to maximize each of sensitivity and specificity, and to provide the best balance between sensitivity and specificity. These search strategies for retrieving high-quality original treatment, diagnosis, and prognosis studies were used in this study [see Additional file 1].

**Figure 1:**
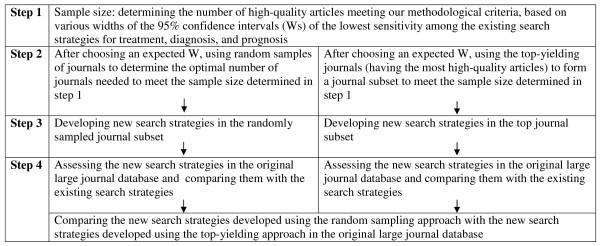
Summary of the main methods used for this project.

The main methods for this project are summarized as 4 steps in figure 1.

### Sample size calculation (Step 1 in figure 1)

The sample size calculation for the number of pass articles needed in a journal subset was based on the desired width of the 95% confidence intervals (W) around the sensitivity of the existing search strategies [16]. We calculated sample sizes based on W's ranging from 0.01 to 0.20.

Sensitivity and specificity (defined above) are 2 key attributes of a search strategy. The maximal W for the specificity among the existing 3 search strategy types (highly sensitive search, highly specific search, and balanced search) was smaller than the minimal W for the sensitivity for each of the treatment, diagnosis, and prognosis categories. For example, for the treatment category, the Ws for the 3 specificities were 0.0028, 0.0039, and 0.0082; the Ws for the 3 sensitivities were 0.0088, 0.0198, and 0.0249. Choosing the sensitivity of the existing search strategies to estimate and calculate the sample size of pass articles would guarantee a high level of specificity. Additionally, because all sensitivities from the 3 existing search strategy types were > 50%, the lowest sensitivity from the high specificity strategy was used as the parameter for this evaluation to guarantee the sample sizes for the other 2 search strategy types (i.e., high sensitivity and balanced combination) based on binomial theory. The lowest sensitivities used in the analysis were 93.1% for the treatment category, 64.4% for diagnosis, and 52.3% for prognosis [see Additional file 1]. We assumed that the distribution of the number of the pass articles was approximately normal. For example, to achieve a W of 0.05 for the treatment category, at least n pass articles are needed in a journal subset to update search strategies in the future, where n is calculated using the formula:

(1) $$\text{n}=\frac{1.96^{2}\times sensitivity\times (1-sensitivity)}{{\left(W/2\right)}^{2}}$$

Therefore, n = [1.96^2 ^× 93.1% × (1 - 93.1%)]/[(0.05/2)^2^] = 395.

### Small journal subsets (Step 2 in Figure 1)

A computer program was developed to create journal subsets by randomly selecting journals from the original 161-journal database. Journal subsets that included ≤ 110 journals were gradually and arbitrarily increased by 5 journals (i.e., subsets were created with 5, 10, 15, 20, and so on up to 110 journals) and journal subsets that included > 110 journals were increased arbitrarily by 10 journals. Thus, 26 subsets of journals were randomly created from the 161-journal database. Each journal had the same probability of selection. We presumed that selected journal subsets were independent, and the same journal might appear in more than 1 journal subset. After the creation of the 26 journal subsets, the number of high-quality articles in each subset was counted.

The 161 journals were also ordered according to the ascending number of pass articles for the treatment [see Additional file 2], diagnosis, and prognosis categories.

Based on an arbitrarily chosen W of 0.10, we determined the optimal journal subset which included the minimal number of high-quality studies that could be used to develop search strategies. The optimal journal subset was formed by 2 approaches – random sampling of journals and top journals.

### Determining the new search strategies (Step 3 in figure 1)

To assess whether the 2 optimal journal subsets formed by random sampling and top journal approaches were acceptable for updating the search strategies in MEDLINE, new search strategies were developed in these 2 journal subsets. We used the same method to developing search strategies that was used previously; 3869 unique search terms were tested in each journal subset for their ability to retrieve high-quality articles of a certain category (e.g., original treatment studies).

### Testing the new search strategies in the original large journal database (Step 4 in figure 1)

The new search strategies derived in the small journal subsets (random sampling and top journal approaches) were assessed in the original large journal database, and compared with the existing search strategies.

If these new search strategies performed poorly (defined as a sensitivity or specificity < 50%) in the original large database, another journal subset with a total number of pass articles to achieve a smaller W (e.g., 0.05) would be assessed.

The chi-squared test (STATA 9.0) was used to compare the performance characteristics between 2 independent journal subsets. A 2-sided significance level of α = 0.05 was adopted.

## Results

### Sample size calculation

Based on the equation noted earlier (1), the sample size requirements for different Ws for the treatment, diagnosis, and prognosis categories are shown in Table 1. For instance, to achieve a W of 0.20, a journal subset with at least 25 pass articles for the treatment category would be needed to update search strategies; for the diagnosis and prognosis categories, journal subsets with at least 88 and 96 pass articles are needed, respectively.

**Table 1:** Required numbers of pass articles for different widths of 95% confidence intervals (Ws) for treatment, diagnosis, and prognosis categories

**Category**	**W (required number of pass articles)**									
Treatment	0.01 (9871)	0.02 (2468)	0.03 (1097)	0.04 (617)	0.05 (395)	0.06 (275)	0.07 (201)	0.08 (155)	0.09 (122)	0.10 (99)
Diagnosis	0.01 (35141)	0.02 (8786)	0.03 (3905)	0.04 (2197)	0.05 (1406)	0.06 (977)	0.07 (717)	0.08 (550)	0.09 (434)	0.10 (352)
Prognosis	0.01 (38335)	0.02 (9584)	0.03 (4259)	0.04 (2396)	0.05 (1534)	0.06 (1065)	0.07 (782)	0.08 (599)	0.09 (473)	0.10 (384)

**Category**	**W (required number of pass articles)**									

Treatment	0.11 (82)	0.12 (69)	0.13 (58)	0.14 (51)	0.15 (44)	0.16 (39)	0.17 (34)	0.18 (31)	0.19 (27)	0.20 (25)
Diagnosis	0.11 (290)	0.12 (245)	0.13 (208)	0.14 (180)	0.15 (156)	0.16 (138)	0.17 (122)	0.18 (109)	0.19 (97)	0.20 (88)
Prognosis	0.11 (317)	0.12 (267)	0.13 (227)	0.14 (196)	0.15 (170)	0.16 (150)	0.17 (133)	0.18 (119)	0.19 (106)	0.20 (96)

### Determining the optimal journal subsets

26 subsets of journals were randomly created from the 161-journal database, and the Ws achieved with the corresponding numbers of pass articles for the 3 purpose categories are shown in Table 2.

**Table 2:** Subsets of randomly sampled journals from the 161-journal database with their corresponding widths of 95% confidence intervals (Ws) achieved and numbers of pass articles for treatment, diagnosis, and prognosis categories

**Number of journal in each randomly sampled subset**	**W (numbers of pass articles in each journal subset)**		
	
	**Treatment**	**Diagnosis**	**Prognosis**
161	0.04 (1587)	0.16 (147)	0.16 (190)
150	0.04 (1483)	0.18 (131)	0.16 (179)
140	0.04 (1425)	0.16 (136)	0.16 (159)
130	0.04 (1154)	0.20 (107)	0.18 (145)
120	0.04 (1200)	0.18 (109)	0.16 (150)
110	0.04 (1228)	0.20 (94)	0.16 (153)
105	0.04 (1027)	0.20 (93)	0.20 (110)
100	0.04 (1019)	0.20 (86)	0.18 (133)
95	0.04 (818)	0.20 (94)	0.20 (108)
90	0.04 (962)	0. 24 (66)	0.22 (79)
85	0.04 (1014)	0.20 (83)	0.18 (128)
80	0.04 (847)	0.22 (70)	0.18 (125)
75	0.04 (632)	0.22 (53)	0.22 (73)
70	0.05 (402)	0.24 (61)	0.24 (69)
65	0.05 (582)	0.26 (54)	0.22 (86)
60	0.04 (660)	0.22 (72)	0.24 (72)
55	0.04 (675)	0.30 (38)	0.24 (66)
50	0.04 (696)	0.30 (38)	0.22 (80)
45	0.05 (395)	0.40 (21)	0.36 (30)
40	0.06 (374)	0.30 (39)	0.28 (51)
35	0.08 (222)	0.38 (24)	0.38 (26)
30	0.08 (263)	0.38 (24)	0.40 (24)
25	0.08 (251)	0.30 (40)	0.34 (35)
20	0.10 (141)	0.36 (28)	0.42 (22)
15	0.08 (191)	0.56 (11)	0.40 (25)
10	0.12 (85)	0.56 (11)	0.50 (15)
5	0.18 (37)	NA* (0)	NA* (1)

*NA = not available.

A W of 0.10 was chosen as a starting point to estimate if a small journal subset was good enough to update search strategies for use in MEDLINE in the future. Based on Table 1, the optimal journal subset must have ≥ 99 pass articles to reach a W of 0.10 for the treatment category. The probabilities of randomly sampling 10, 15, and 20 journals to achieve a W of 0.10 are 63.56%, 94.04%, and 99.63%, respectively [see Additional file 3]. Therefore, a subset of 15 randomly sampled journals that has a probability of 94% to achieve a W ≤ 0.10 seems to be the optimal and most efficient journal subset to use when updating search strategies for retrieving treatment studies in the future.

The 2 top-yielding journals (*The Lancet *and *Journal of Clinical Oncology*) included 158 pass articles (i.e., > 99) in the treatment category and could also guarantee a W ≤ 0.10. Thus, just 2 top journals could be used when updating treatment search strategies.

### Developing new search strategies for treatment using the small journal subsets

New search strategies (3 types: high sensitivity, high specificity, and balanced combination of sensitivity and specificity) were developed in 1 subset of 15 randomly sampled journals [see Additional file 4] and are shown in Table 3. Similarly, new search strategies were developed in the subset of 2 top journals and are shown in Table 3 as well.

**Table 3:** Comparing the new strategies from 15 randomly sampled journals (R15) and from 2 top-yielding journals (T2) with the existing search strategies (ES) in the original large journal database

Ovid search strategy*		**Sensitivity (%) (95% CI)**	**Specificity (%) (CI)**	**Precision (%) (CI)**	**Accuracy (%) (CI)**
**High sensitivity**	**ES: **clinical trial.mp, pt. OR random:.mp. OR tu.xs.	99.2 (98.7, 99.8)	70.4 (69.8, 70.9)	9.9 (9.3, 10.5)	71.3 (70.8, 71.8)
	**R15: **clinical trial.mp, pt. OR random:.mp. OR between group:.tw.	98.5 (97.7, 99.3)	76.3‡ (75.8, 76.8)	11.9‡ (11.2, 12.7)	77.0‡ (76.5, 77.4)
	**T2: **clinical trial.mp, pt. OR exp longitudinal studies.	96.8† (95.6, 97.9)	84.1‡ (83.7, 84.6)	16.6‡ (15.6, 17.6)	84.5‡ (84.1, 84.9)

**High specificity**	**ES: **randomized controlled trial.mp, pt.	93.1 (91.5, 94.8)	97.5 (97.3, 97.6)	54.4 (52.0, 56.8)	97.3 (97.1, 97.5)
	**R15: **double-blind.mp. OR random: assigned.tw.	47.1† (43.9, 50.3)	98.8‡ (98.7, 99.0)	57.0 (53.5, 60.5)	97.2 (97.0, 97.4)
	**T2: **double-blind:.mp. OR random: assigned.tw.	53.4† (50.2, 56.7)	98.4‡ (98.3, 98.6)	52.7 (49.5, 55.8)	97.0† (96.8, 97.2)

**Balanced combination of sensitivity**	**ES: **randomized controlled trial.pt. OR randomized.mp. OR placebo.mp.	95.8 (94.5, 97.1)	95.0 (94.8, 95.3)	38.5 (36.5, 40.5)	95.0 (94.8, 95.3)
	**R15: **randomized controlled trial.pt. OR random: assigned.tw. OR exp research design.	95.2 (93.8, 96.5)	94.6† (94.3, 94.8)	36.3 (34.4, 38.2)	94.6† (94.3, 94.8)
	**T2: **randomized controlled trial.mp, pt. OR random: assigned.tw. OR blind:.mp.	95.4 (94.0, 96.7)	96.1‡ (95.9, 96.3)	44.3‡ (42.2, 46.5)	96.1‡ (95.8,96.3)

*mp = multiple postings, search term appears in title, abstract or subject heading; pt = publication type; : = truncation; tu = therapeutic use subheading; xs = exploded subheading; tw = textword; exp = explosion.

† Statistically significant difference (p-value < 0.05) in favor of the existing search strategy.

‡ Statistically significant difference (p-value < 0.05) in favor of the new search strategy.

### Comparing the new search strategies for treatment with the existing search strategies in the original large journal database

The new search strategies for the treatment category that were developed in the subset of 15 randomly sampled journals were tested in the original large journal database and the performance was compared with the existing search strategies (Table 3). When comparing the high sensitivity search strategies, the sensitivities were not significantly different (98.5% versus 99.2%); the specificity, precision, and accuracy of the new strategy, however, were statistically higher than those of the existing strategy. When comparing the high specificity search strategies, the specificity of the new strategy was 1.3% higher than that of the existing search strategy (98.8% versus 97.5%, p-value < 0.001); the sensitivity (47.1%, 95% CI 43.9% to 50.3%), however, was much lower than that of the existing strategy (93.1%, 95% CI 91.5% to 94.8%); the precision and accuracy did not differ statistically. When comparing the strategies for the balanced combination of sensitivity and specificity, the sensitivities (95.2% versus 95.8%) were not significantly different; the specificity (94.6%) of the new strategy was similar to that of the existing search strategy (95.0%), but the difference was significant (p-value = 0.032).

Based on the above analysis, except for the sensitivity of the new high specificity search strategy being lower than that of the existing search strategy, the performance characteristics of the new strategies derived using the 15 randomly sampled journals appeared to be as good as those of the existing search strategies. The new high specificity strategy ("double-blind.mp." OR "random: assigned.tw.") and the existing high specificity strategy ("randomized controlled trial.mp, pt.") retrieved 146,416 and 259,665 eligible articles, respectively, from an Ovid MEDLINE search (conducted on May 24, 2008). Compared with the existing strategy, the new specificity strategy would save about 44% of the time to read the eligible articles. However, this strategy would miss about 57,801 (41%) relevant articles, some of which might be important.

In Table 3, the new search strategies developed using the subset of 2 top journals were also compared with the existing search strategies in the original large journal database. Similarly, the performance characteristics of the new strategies appeared to be as good as those of the existing search strategies except for the sensitivity (53.4%) of the new high-specificity strategy. In all cases, the choice of search strategy type depends on the end user's need.

### Comparing the 2 new strategies for treatment

The strategies developed using the subset of 15 randomly selected journals and the 2 top journals were compared in the original large journal database (Table 3 – data not shown for the comparison). The 2 new strategies seemed to work well except for the sensitivities from the high specificity search strategies. Both new high sensitivity strategies yielded good sensitivities (98.5% [CI 97.7, 99.3] versus 96.8% [95.6, 97.9]), but their difference was statistically significant, in favor of the random sampling approach (p-values = 0.016); as a trade-off, the strategy from the random approach yielded lower specificity (76.3% versus 84.1%, p-value < 0.001). For the balanced combination search strategies, the sensitivities derived from the 2 new strategies were similar (95.2% versus 95.4%, p-value = 0.839); the specificity from the random sampling approach was a little lower than that from the top journal approach (94.6% versus 96.1%, p-value < 0.001).

Overall, the top journal approach will be more efficient in future research than the random sampling approach because it requires fewer journals to achieve a required W. However, the strategies from the top journal approach might not perform well in a low-yielding journal subset. This phenomenon may be due to "clustering effects" – the journals in the top journal subset not only have a relatively high proportion of pass articles but also these journals may have other relevant features that aid retrieval such as better writing by authors, editing by publishers, and indexing by bibliographic database providers.

### Testing the 2 new strategies for treatment in a low-yielding journal subset

To test the above hypothesis, the new strategies from the random sampling approach were tested in a low-yielding journal subset that included 103 journals from the 161-journal database, in which each journal had 0 to 5 pass articles on treatment. The total number of pass articles in the low-yielding journal subset (192) was almost equal to that in the subset of 15 randomly selected journals (191). Similarly, the new strategies from the top journal approach were tested in a low-yielding journal subset that included 97 journals with 158 pass articles that was equal to that in the 2 top journals. Almost all search strategies developed using the top journal approach performed less well in the low-yielding journal subset [see Additional file 5], while the new search strategies developed using the random sampling approach performed better in the low-yielding journal subset on some tests [see Additional file 6]. For instance, the sensitivity of the high sensitivity strategy was higher in the subset of 103 low-yielding journals than that in the subset of 15 randomly sampled journals (100.0% vs. 98.4%), as was the specificity of the balanced combination strategy (95.2% vs. 95.1%).

### Diagnosis and prognosis categories

For both the diagnosis and prognosis categories, even when using the 161-journal database, the smallest W achieved was 0.16, because the numbers of pass articles were very low, 147 (0.30% among 49,028 articles) for diagnosis and 190 (0.39%) for prognosis, compared with 1587 (3.24%) for treatment category. If we accept a wider W, such as a W = 0.20, we could find a smaller journal subset for diagnosis and prognosis based on the sample size calculation for the number of the pass articles in Table 1. If we pursue a narrow W, such as a W = 0.10, we need to hand search > 161 journals in order to identify the required number of the high-quality articles for diagnosis (≥ 352) and for prognosis (≥ 384). That would be expensive and time-consuming.

## Discussion

The sample size calculations shown in this study suggest that search strategies developed in small journal subsets will be as good as those developed in larger collections of journals if there are a sufficient number of high-quality articles. In this case, the subsets of 15 randomly sampled journals or 2 top-yielding journals that included ≥ 99 high-quality articles achieved a W of 0.10 for the retrieval of treatment studies. Except for the sensitivities of the high specificity search strategies, the other performance characteristics of the new strategies developed using both the random sampling and top journal approaches were close to those of the existing search strategies (Table 3). The sensitivities were > 95% for both the high sensitivity strategies and balanced combination strategies derived using the 2 approaches. If the end users have enough time and concern for retrieving all the high-quality studies, they could choose either of these search strategies.

This study has some limitations. First, the new search strategies developed using the random sampling approach were done using a subset of 15 randomly sampled journals with ≥ 99 pass articles. As shown earlier, there is a 6% probability [see Additional file 3] that another subset of 15 randomly sampled journals will have < 99 pass articles. In future research, if a subset of 15 randomly sampled journals has < 99 pass articles, we need to gradually add journals that are randomly selected one by one until the total number of the pass articles in the journal subset is ≥ 99. Second, the high performance search strategies developed using each subset of 15 randomly sampled journals that includes ≥ 99 pass articles may be slightly different even though each subset has a similar number of pass articles. This is the case because no 2 pass articles have identical content and it is unlikely that 2 similar articles would have exactly the same index terms. Nevertheless, it does not matter whether the search terms are the same, as long as search performances are equivalent. This is most likely the case because as we found in our previous research many different search strategies had very similar performance. Third, the concentrations of diagnosis and prognosis articles were too low to update or create new search strategies using either approach, random sampling or top journal.

The new search strategies developed using the random sampling approach seem to perform better than the new strategies developed using the top journal approach in low-yielding journals. This is not surprising because the subsets of randomly sampled journals included both top-yielding and low-yielding journals.

## Conclusion

The search strategies that are widely used by clinicians, health researchers, and librarians in the Clinical Queries interface of PubMed and in Ovid were developed in journals published in 2000 and will need to be updated periodically to maintain and improve their performance as well as to address new topic areas. When updating or creating new search strategies for high-quality articles on therapy in MEDLINE in future research, the approach of randomly sampling a subset of journals that includes sufficient high-quality articles provides the most parsimonious way of achieving performance estimates at a specified level of statistical precision. For treatment studies, the number of journals needed is quite small because the concentration of high-quality studies is quite high in clinical journals. For diagnosis and prognosis articles, however, the concentration of high-quality studies is low, and a large number of journals are needed for the development of search strategies. The expense of developing and testing search strategies is high and this research provides a way to estimate how much work will be needed to achieve a robust result.

## Abbreviations

W: width of the 95% confidence intervals.

## Competing interests

The authors declare that they have no competing interests.

## Authors' contributions

XY designed the research, analyzed and interpreted data, and wrote the manuscript. NLW contributed to the analysis of data and revised the manuscript. SDW contributed to the interpretation of data and revised the manuscript. RBH designed the research and revised the manuscript.

## Pre-publication history

The pre-publication history for this paper can be accessed here:



## Supplementary Material

Additional file 1 Click here for file

Additional file 2 Click here for file

Additional file 3 Click here for file

Additional file 4 Click here for file

Additional file 5 Click here for file

Additional file 6 Click here for file
