# Supplementary material for: Sample size determination for bibliographic retrieval studies
Source: BMC Med Inform Decis Mak. 2008 Sep 29;8:43. doi: 10.1186/1472-6947-8-43 (PMC2569926; doi:10.1186/1472-6947-8-43)
Supplement: Additional file 1 [file 1472-6947-8-43-S1.pdf]

Additional file 1 - Best search strategies and retrieval performance for treatment, diagnosis, and prognosis from 161 journals in MEDLINE in 2000 [7-9]

Category	Strategy type	Ovid search strategy*	Sen/Spe/Pre/Acc (%)†
**Treatment**	**High sensitivity**	clinical trial.mp. OR clinical trial.pt. OR random:.mp. OR tu.xs.	99.2/70.4/9.9/71.3
	**High specificity**	randomized controlled trial.mp,pt.	93.1‡/97.5/54.4/97.3
	**Balanced combination of sensitivity & specificity**	randomized controlled trial.pt. OR randomized.mp. OR placebo.mp.	95.8/95.0/38.5/95.0
**Diagnosis**	**High sensitivity**	Sensitive:.mp. OR diagnos:.mp. OR di.fs.	98.6/74.3/1.1/74.3
	**High specificity**	Specificity.tw.	64.6‡/98.4/10.6/98.3
	**Balanced combination of sensitivity & specificity**	Sensitive:.mp. OR predictive value:.mp. OR accuracy:.tw.	92.5/92.1/3.4/92.1
**Prognosis**	**High sensitivity**	Incidence.sh. OR exp mortality OR follow-up studies.sh. OR prognos:.tw OR predict:.tw. OR course:.tw.	90.1/79.7/1.7/79.7
	**High specificity**	prognos:.tw. OR first episode.tw. OR cohort.tw.	52.3‡/94.1/3.3/93.9
	**Balanced combination of sensitivity & specificity**	prognosis.sh. OR diagnosed.tw. OR cohort:.mp. OR predictor:.tw. OR death.tw. OR exp models, statistical	82.9/83.7/1.9/83.7

*mp = multiple posting (term in title, abstract, or MeSH heading); pt = publication type; tu = therapeutic use subheading; : = truncation; xs = exploded subheading; di = diagnosis subheading; fs = floating subheading; tw = textword; sh = subject heading MeSH; exp = explosion.

†Sen = Sensitivity; Spe = Specificity; Pre = Precision; Acc = Accuracy.

‡The lowest sensitivity among the 3 strategy types for each category of article.
