# Supplementary material for: Sample size determination for bibliographic retrieval studies
Source: BMC Med Inform Decis Mak. 2008 Sep 29;8:43. doi: 10.1186/1472-6947-8-43 (PMC2569926; doi:10.1186/1472-6947-8-43)
Supplement: Additional file 2 [file 1472-6947-8-43-S2.pdf]

Additional file 2 - 161-journal list ordered by descending number of pass articles (N_P) for the treatment category

Journal number	Journal name	N_P for treatment	N_P for prognosis	N_P for diagnosis
J-129	LANCET	87	3	1
J-102	J CLIN ONCOL	71	9	0
J-56	CIRCULATION	71	1	2
J-133	N ENGL J MED	66	5	3
J-88	J AM COLL CARDIOL	52	10	3
J-7	AM J CARDIOL	50	3	4
J-64	CRIT CARE MED	45	1	1
J-128	JAMA	43	3	2
J-66	DIABETES CARE	38	0	1
J-134	NEUROLOGY	37	8	4
J-137	OBSTET GYNECOL	37	6	9
J-39	BMJ	37	1	2
J-11	AM J OBSTET GYNECOL	36	7	3
J-54	CHEST	35	1	5
J-28	ARCH INTERN MED	34	4	2
J-70	FERTIL STERIL	34	0	2
J-14	AM J RESPIR CRIT CARE MED	32	4	2
J-141	PEDIATRICS	29	9	3
J-112	J INFECT DIS	28	10	0
J-42	BR J OBSTET GYNAECOL	27	0	2
J-9	AM J GASTROENTEROL	26	1	0
J-157	STROKE	23	13	0
J-18	ANN INTERN MED	23	0	0
J-104	J CLIN PSYCHOPHARMACOL	21	0	0
J-105	J CONSULT CLIN PSYCHOL	20	0	1
J-120	J PEDIATR	19	9	3
J-125	J RHEUMATOL	19	3	2
J-52	CANCER	17	7	0
J-44	BR J SURG	17	1	0
J-90	J AM GERIATR SOC	17	1	0
J-27	ARCH GEN PSYCHIATRY	16	1	2
J-156	SPINE	16	1	0
J-21	ANN SURG	16	0	1
J-103	J CLIN PSYCHIATRY	15	1	0
J-10	AM J MED	14	3	1
J-77	HEART	14	2	1
J-17	ANN EMERG MED	14	1	3
J-32	ARCH PHYS MED REHAB	13	3	1
J-25	ARCH DIS CHILD FETAL NEO ED	13	3	0
J-12	AM J PSYCHIATRY	13	2	1
J-159	THORAX	13	0	2

J-26	ARCH FAM MED	12	0	1
J-43	BR J PSYCHIATRY	10	5	1
J-74	GUT	10	4	1
J-85	J AFFECTIVE DISORD	10	3	0
J-67	DIABETIC MED	10	1	2
J-16	AM J SURG	10	0	2
J-72	GASTROENTEROLOGY	10	0	1
J-138	PAIN	10	0	0
J-1	ACTA OBSTET GYNECOL SCAND	9	0	2
J-2	ACTA ORTHOPAED SCAND	9	0	0
J-34	ARTHRITIS RHEUM	7	2	1
J-59	CLIN ORTHOP RELATED RES	7	2	0
J-33	ARCH SURG	7	0	0
J-35	ARTHROSCOPY	7	0	0
J-127	J VASC SURG	7	0	0
J-24	ARCH DIS CHILD	6	4	4
J-20	ANN RHEUMATIC DIS	6	2	0
J-3	ACTA PSYCHIATR SCAND	6	0	1
J-4	ADDICTION	6	0	1
J-92	J ARTHROPLASTY	6	0	0
J-95	J BONE JOINT SURG BR	6	0	0
J-115	J NEUR NEUROSURG PSYCHIATRY	5	2	3
J-41	BR J GEN PRACT	5	0	2
J-108	J FAM PRACT	5	0	1
J-79	HYPERTENSION	5	0	0
J-114	J MANIPULATIVE PHYSIOL THER	5	0	0
J-136	NURS RES	5	0	0
J-31	ARCH PED ADOL MED	4	3	2
J-86	J AM ACAD CHILD ADOL PSYCHIATR	4	2	1
J-76	HEALTH PSYCHOL	4	2	0
J-97	J CHILD PSYCHOL PSYCHIATRY	4	2	0
J-111	J HAND SURG (BR)	4	1	1
J-124	J PSYCHOSOM RES	4	1	1
J-126	J TRAUMA	4	0	3
J-117	J NEUROSURG and J NEUROSURG SPINE	4	0	1
J-5	AGE AGEING	4	0	0
J-37	BEHAV RES THER	4	0	0
J-96	J CHILD ADOL PSYCHOPHARM	4	0	0
J-109	J GEN INTERN MED	4	0	0
J-158	SURGERY	4	0	0
J-152	RADIOLOGY	3	1	13
J-81	INT J GERIATR PSYCHIATRY	3	1	6
J-60	CLIN PEDIATR	3	1	1

J-83	J ABNORM PSYCHOL	3	1	0
J-130	MED CARE	3	1	0
J-62	CMAJ	3	0	1
J-131	MED J AUST	3	0	1
J-47	CAN J NURS RES	3	0	0
J-48	CAN J PSYCHIATRY	3	0	0
J-51	CAN RESPIR J	3	0	0
J-53	CANCER NURS	3	0	0
J-94	J BONE JOINT SURG AM	3	0	0
J-15	AM J SPORTS MED	2	1	1
J-30	ARCH NEUROL	2	1	0
J-142	PLASTIC RECON SURG	2	1	0
J-6	AJR	2	0	11
J-147	PSYCHOL MED	2	0	2
J-148	PSYCHOSOM MED	2	0	2
J-38	BIRTH	2	0	0
J-45	CAN J CARDIOL	2	0	0
J-46	CAN J GASTROENTEROL	2	0	0
J-55	CHILD DEV	2	0	0
J-75	HEALTH EDUC BEHAV	2	0	0
J-78	HEART LUNG	2	0	0
J-82	J ABNORM CHILD PSYCHOL	2	0	0
J-89	J AM COLL SURG	2	0	0
J-139	PATIENT EDUC COUNS	2	0	0
J-99	J CLIN EPIDEMIOL	1	0	2
J-98	J CLIN CHILD PSYCHOL	1	0	1
J-123	J PEDIATR ORTHOPEDICS	1	0	1
J-13	AM J PUBLIC HEALTH	1	0	0
J-23	APPL NURS RES	1	0	0
J-36	AUST N Z J PSYCHIATRY	1	0	0
J-49	CAN J PUBLIC HEALTH	1	0	0
J-57	CLIN INVEST MED	1	0	0
J-65	DEV PSYCHOPATHOL	1	0	0
J-69	FAM PRACTICE	1	0	0
J-84	J ADV NURS	1	0	0
J-91	J AM MED INFORM ASSOC	1	0	0
J-100	J CLIN EXP NEUROPSYCHOL	1	0	0
J-107	J EPIDEMIOL COMMUNITY HEALTH	1	0	0
J-116	J NEUROPSYCH CLIN NEUROSCI	1	0	0
J-118	J NURS SCHOLAR	1	0	0
J-144	PSYCHOL AGING	1	0	0
J-153	RES NURS HEALTH	1	0	0
J-160	WEST J MED	1	0	0
J-161	WEST J NURS RES	1	0	0
J-8	AM J EPIDEMIOL	0	4	0

J-113	J INTERN MED	0	2	0
J-143	PSYCHIATR SERV	0	1	0
J-154	SCHIZOPHR BULL	0	1	0
J-154	SCHIZOPHR BULL	0	1	0
J-154	SCHIZOPHR BULL	0	1	0
J-145	PSYCHOL ASSESS	0	0	2
J-50	CAN J SURG	0	0	1
J-110	J HAND SURG (AM)	0	0	1
J-140	PEDIATR RADIOL	0	0	1
J-19	ANN MED	0	0	0
J-22	ANS ADV NURS SCI	0	0	0
J-29	ARCH MED RES	0	0	0
J-40	BR J CLIN PSYCHOL	0	0	0
J-58	CLIN NURS RES	0	0	0
J-61	CLIN PSYCHOL REV	0	0	0
J-63	COCHRANE LIBRARY	0	0	0
J-68	FAM PLAN PERSPECT	0	0	0
J-71	FOOT ANKLE INTERN	0	0	0
J-73	GEN HOSP PSYCHIATRY	0	0	0
J-80	INJURY	0	0	0
J-87	J AM BOARD FAM PRACT	0	0	0
J-93	J AUTISM DEV DISORD	0	0	0
J-101	J CLIN NURS	0	0	0
J-106	J CUTAN MED SURG	0	0	0
J-119	J ORTHOPAEDIC RES	0	0	0
J-121	J PEDIATR NURS	0	0	0
J-122	J PEDIATR ONCOL NURS	0	0	0
J-132	MIDWIFERY	0	0	0
J-135	NEUROSURG	0	0	0
J-146	PSYCHOL BULL	0	0	0
J-149	PUBLIC HEALTH NURS	0	0	0
J-150	QUAL HEALTH CARE	0	0	0
J-151	QUAL HEALTH RES	0	0	0
J-155	SOC SCI MED	0	0	0
