# Supplementary material for: Sample size determination for bibliographic retrieval studies
Source: BMC Med Inform Decis Mak. 2008 Sep 29;8:43. doi: 10.1186/1472-6947-8-43 (PMC2569926; doi:10.1186/1472-6947-8-43)
Supplement: Additional file 3 [file 1472-6947-8-43-S3.pdf]

Additional file 3 - Probabilities for subsets of any numbers of randomly sampled journals with ≥ 99 pass articles

Journal subsets	Average probability (%)	SE* of probability (%)
1	0	0
2	1.01	0.01
3	3.67	0.02
4	8.06	0.02
5	14.98	0.03
6	23.26	0.04
7	32.87	0.04
8	43.23	0.04
9	53.64	0.04
10	63.56	0.043
11	72.32	0.04
12	79.89	0.04
13	85.95	0.03
14	90.51	0.03
15	94.04	0.02
16	96.29	0.02
17	97.74	0.01
18	98.66	0.01
19	99.22	0.01
20	99.63	0.01
21	99.82	0.00
22	99.90	0.00
23	99.95	0.00
24	99.98	0.00
25	99.99	0.00
26	100.00	0.00
27	100.00	0.00

*SE = standard error.
