# Supplementary material for: Sample size determination for bibliographic retrieval studies
Source: BMC Med Inform Decis Mak. 2008 Sep 29;8:43. doi: 10.1186/1472-6947-8-43 (PMC2569926; doi:10.1186/1472-6947-8-43)
Supplement: Additional file 4 [file 1472-6947-8-43-S4.pdf]

Additional file 4 - A subset of 15 randomly sampled journals from the 161-journal database in MEDLINE*

**Journal number**	**Journal name**
1	ACTA PSYCHIATR SCAND
2	ANN RHEUMATIC DIS
3	BEHAV RES THER
4	BR J SURG
5	HEALTH PSYCHOL
6	J AM BOARD FAM PRACT
7	J CLIN EXP NEUROPSYCHOL
8	J CUTAN MED SURG
9	J PEDIATR
10	J TRAUMA
11	J VASC SURG
12	LANCET
13	NEUROLOGY
14	NURS RES
15	PUBLIC HEALTH NURS

*Including 191 pass article for the treatment category.
