# Supplementary material for: Sample size determination for bibliographic retrieval studies
Source: BMC Med Inform Decis Mak. 2008 Sep 29;8:43. doi: 10.1186/1472-6947-8-43 (PMC2569926; doi:10.1186/1472-6947-8-43)
Supplement: Additional file 5 [file 1472-6947-8-43-S5.pdf]

Additional file 5 - Testing the new strategies developed using 2 top journals (T2J) in a low-yielding journal (LJ) subset*

Ovid search strategy†		Sensitivity (%) (95% CI)	Specificity (%) (CI)	Precision (%) (CI)	Accuracy (%) (CI)
**High sensitivity** clinical trial.mp,pt. OR exp longitudinal studies	**LJ subset**	91.8‡ (87.5, 96.1)	85.1‡ (84.5, 85.8)	7.7‡ (6.5, 8.9)	85.2‡ (84.6, 85.8)
	**T2J subset**	100 (100, 100)	87.3 (86.1, 88.5)	29.6 (25.8, 33.5)	87.9 (86.8, 89.1)
**High specificity** double-blind.mp. OR random: assigned.tw.	**LJ subset**	42.4 (34.7, 50.1)	98.9 (98.7, 99.1)	34.0‡ (27.4, 40.6)	98.1 (97.9, 98.4)
	**T2J subset**	53.2 (45.4, 60.9)	99.2 (98.8, 99.5)	77.1 (69.2, 85.0)	96.8 (96.2, 97.4)
**Balanced optimization of sensitivity & specificity** randomized controlled trial.mp,pt. OR random: assigned.tw. OR blind:.mp.	**LJ subset**	89.9‡ (85.2, 94.6)	96.6‡ (96.3, 97.0)	26.5‡ (22.8, 30.3)	96.6‡ (96.2, 96.9)
	**T2J subset**	98.1 (96.0, 100.0)	97.7 (97.2, 98.3)	69.8 (63.8, 75.9)	97.8 (97.2, 98.3)

*Both of 2 top journals and the low-yielding journal subset have 158 pass articles.
†mp = multiple postings, search term appears in title, abstract or subject heading; pt = publication type; : = truncation; tw = textword; exp = explosion.
‡The performance of the new strategies is statistically significantly (p-value < 0.05) lower in the low-yielding journal subset than in the 2 top journals.
