# Supplementary material for: Sample size determination for bibliographic retrieval studies
Source: BMC Med Inform Decis Mak. 2008 Sep 29;8:43. doi: 10.1186/1472-6947-8-43 (PMC2569926; doi:10.1186/1472-6947-8-43)
Supplement: Additional file 6 [file 1472-6947-8-43-S6.pdf]

Additional file 6 - Testing the new strategies developed using the subset of 15 randomly selected journals (R15J) in a low-yielding journal (LJ) subset*

Ovid search strategy†		Sensitivity (%) (95% CI)	Specificity (%) (CI)	Precision (%) (CI)	Accuracy (%) (CI)
**High sensitivity** clinical trial.mp, pt. OR random:.mp. OR between group:.tw.	**LJ subset**	100.0‡ (100.0, 100.0)	76.0 (75.4, 76.7)	4.4 (3.8, 5.0)	76.3 (75.7, 76.9)
	**R15J subset**	98.4 (96.7, 100)	80.1 (79.1, 81.1)	13.8 (12.0, 15.7)	80.7 (79.7, 81.7)
**High specificity** double-blind.mp. OR random: assigned.tw.	**LJ subset**	44.3 (37.2, 51.3)	99.1 (98.9, 99.2)	34.4 (28.5, 40.3)	98.5‡ (98.3, 98.7)
	**R15J subset**	59.7 (52.7, 66.7)	99.1 (98.8, 99.3)	67.5 (60.4, 74.5)	97.8 (97.5, 98.2)
**Balanced optimization of sensitivity & specificity** randomized controlled trial.pt. OR random: assigned.tw. OR exp research design	**LJ subset**	92.7 (89.0, 96.4)	95.2‡ (94.9, 95.5)	17.6 (15.2, 19.9)	95.2‡ (94.9, 95.5)
	**R15J subset**	94.8 (91.6, 97.9)	95.1 (94.5, 95.6)	38.3 (34.0, 42.7)	95.1 (94.5, 95.6)

*The subset of 15 randomly selected journals has 191 pass articles; the low-yielding journal subset has 192 pass articles.

†mp = multiple postings, search term appears in title, abstract or subject heading; pt = publication type; : = truncation; tw = textword; exp = explosion.

‡The performance of the new strategies is higher in the low-yielding journal subset than in the subset of 15 randomly selected journals (the p-value could not be calculated because these 2 journal subsets were not independent: some journals were overlapped).
